# The Effect of Removable Space Maintainer Use on Oral-Health-Related Quality of Life in Children: A Cross-Sectional Study

**DOI:** 10.3290/j.ohpd.c_2504

**Published:** 2026-02-10

**Authors:** Fatma Saraç, Fatıma Nuran Kiran, Shokoufeh Ranjbarsisan, Feride Aktaş

**Affiliations:** a Fatma Saraç Paediatric Dentist, Department of Paediatric Dentistry, Atatürk University Faculty of Dentistry, Erzurum, Turkey. Study design and research, conducting the study, writing the manuscript, reviewing and editing the manuscript.; b Fatıma Nuran Kıran Paediatric Dentist, Department of Paediatric Dentistry, Atatürk University Faculty of Dentistry, Erzurum, Turkey. Study design and research, conducting the study, writing the manuscript, reviewing and editing the manuscript.; c; d Shokoufeh Ranjbarsisan Paediatric Dentist, Department of Paediatric Dentistry, Atatürk University Faculty of Dentistry, Erzurum, Turkey. Study design and research, conducting the study, writing the manuscript, reviewing and editing the manuscript.; e Feride Aktaş Paediatric Dentist, Department of Paediatric Dentistry, Atatürk University Faculty of Dentistry, Erzurum, Turkey. Study design and research, conducting the study, writing the manuscript, reviewing and editing the manuscript.

**Keywords:** CPQ8–10, oral-health-related quality of life, pediatric dentistry, removable space maintainer

## Abstract

**Purpose:**

This study aimed to evaluate the oral-health-related quality of life (OHRQoL) of children aged 8–10 years using removable space maintainers (RSMs) and to compare their outcomes with caries-free healthy peers.

**Materials and Methods:**

This cross-sectional study included 51 children who had been using RSMs for at least six months and 51 systemically healthy and caries-free children as controls. All participants were aged 8–10 years. Demographic data were recorded, and the Child Perceptions Questionnaire (CPQ8–10) was administered. The regular use of RSM was determined based on statements from both children and parents. Group comparisons of CPQ8–10 scores were performed using the Mann–Whitney U test and the independent samples t-test, with the level of significance set at P < 0.05.

**Results:**

Children using RSMs had significantly higher total CPQ8–10 scores (26.88 ± 11.94) compared to the control group (12.24 ± 7.31) (P < 0.001). Additionally, RSM users demonstrated significantly higher scores in all subscales, including oral symptoms, functional limitations, emotional well-being, and social well-being (P < 0.05), showing poorer OHRQoL. No significant differences were observed between regular and irregular RSM users in any CPQ8–10 domain (P > 0.05). Gender and age did not significantly affect total CPQ8–10 scores.

**Conclusion:**

The use of RSMs was associated with lower oral-health-related quality of life among children aged 8–10 years when compared with caries-free peers. RSM use could have exerted a deleterious effect on functional limitations, emotional and social well-being. Clinicians may need to consider psychosocial adaptation in addition to clinical indications when planning treatment with RSMs and provide supportive, child-centred approaches during follow-up.

Premature loss of primary teeth due to trauma, caries, or other factors before their natural exfoliation period can lead to a reduction in arch length and various problems related to tooth alignment.^14,16,22^ In a previous study,^15^ early tooth loss in children aged 0–16 years was evaluated. The results showed that 94.7% of the missing teeth were primary teeth, with tooth loss occurring most frequently in mandibular primary molars (37.6%) and maxillary primary molars (31.6%). To avoid such complications, different types of space maintainers, either removable or fixed, can be designed and implemented depending on the number and position of the missing teeth.^19,21,24^ Space maintainer therapy aims to reduce the risk of dental crowding, ectopic eruption, displacement of the successor teeth, and the development of malocclusion.^10,25^ However, both removable and fixed appliances may be difficult for children to tolerate, and they may initially cause discomfort related to speech, mastication, or aesthetics.^18,23^

Oral-health-related treatments in children should be evaluated not only in terms of clinical success but also with regard to their impact on the child’s overall quality of life.^26^ The Child Oral Health-Related Quality of Life Questionnaire (COHQoL) is a comprehensive instrument developed to assess the perceptions of both children and parents concerning oral-health-related quality of life and it has been widely used in the literature.^1,4,11,20^ The scale integrates the viewpoints of the child and the caregiver, which consists of three components: the Parental/Caregiver Perceptions Questionnaire (P-CPQ 17, the Family Impact Scale (FIS)^7^ and the Child Perceptions Questionnaire (CPQ). The CPQ section allows age-specific assessments that take into account the child’s cognitive, emotional, and social developmental stages. This scale was originally developed in Canada by adapting oral-health-related quality of life questionnaires designed for adults to be suitable for children aged 6–14 years. It was later divided into three age-specific versions: CPQ6–7, CPQ8–10 5 and CPQ11–149. The Turkish validity and reliability study of the CPQ8–10 version was conducted by Aydınoğlu et al,^5^ demonstrating that it is suitable for use in evaluations based on children’s self-reported outcomes in the Turkish population.

The CPQ8–10 questionnaire consists of four subscales: oral symptoms, functional limitations, emotional well-being, and social well-being. The CPQ8–10 was chosen for the present study because it enables direct self-reporting from children aged 8–10 years and has been validated in Turkey, providing a reliable measure of oral-health-related quality of life for this age group.

Based on current evidence, no previous study has investigated the effect of removable space maintainers (RSMs) on children’s oral-health-related quality of life. Therefore, the present study aims to compare the oral-health-related quality of life of children using RSMs with that of caries-free children. The hypothesis of this study is that there is no significant difference in quality-of-life scores between children using RSMs and those in the caries-free control group.

## MATERIALS AND METHODS

This cross-sectional study was approved by the Ethics Committee of Atatürk University, Faculty of Dentistry (25 September 2025 – 08/2025) and was conducted in accordance with the principles of the Declaration of Helsinki. Written informed consent was obtained from the parents. Data were collected between September 2025 and October 2025.

Patients who received RSMs composed of an acrylic base and retention elements between 1 January 2024 and 1 February 2025 were identified through archival patient records, and those who agreed to participate were scheduled for clinical examination. Children in both the study and control groups were excluded if dental caries or oral lesions that could potentially influence oral-health-related quality of life were detected. In addition, children with premature primary tooth loss were excluded from the control group. A total of 51 children using RSMs comprised the study group, while systemically healthy children with no active dental caries (up to three restored teeth) comprised the control group.

Children in the control group were selected from patients who presented to the Department of Paediatric Dentistry at Atatürk University Faculty of Dentistry for routine examinations and who exhibited no systemic disease. Children aged 8–10 years were included in both groups.

After recording the children’s age, sex, and whether they used the space maintainer regularly, the CPQ8–10 questionnaire was administered. Regular use of the space maintainer was evaluated based on the statements of both the child and the parent. Children who reported wearing the appliance continuously throughout the day except during meals and toothbrushing, and who attended their follow-up appointments on schedule, were classified as ‘regular users’. Children who reported irregular or inconsistent use of the appliance, including removal during school hours, intermittent or occasional use in response to parental prompting, and/or failure to attend scheduled follow-up appointments, were categorised as ‘irregular users’.

The CPQ8–10 index consists of 25 items divided into four subscales: oral symptoms, functional limitations, emotional well-being, and social well-being. Each item is scored using a five-point Likert scale: never (0), once or twice (1), sometimes (2), often (3), and every day or almost every day (4). The total score ranges from 0 to 100, with higher scores indicating poorer oral-health-related quality of life. Children were instructed to complete the questionnaire based on oral health problems they had experienced within the previous month.

### Sample Size

The expected effect size (Cohen’s d) was taken from a previous study that evaluated changes in OHRQoL after pulpectomy and tooth extraction.^6^ Based on the reported mean difference (4.86 ± 6.13), the calculated effect size was approximately d ≈ 0.8, which corresponds to a large effect size. This value was used in the G*Power software to perform the sample size calculation (two-tailed t-test, α = 0.05, power = 0.95). The required sample size was determined to be 42 participants per group, yielding a total of 84 participants. To minimise potential data loss and ensure sufficient statistical power, 51 participants were included in each group, resulting in a total of 102 participants in the study.

### Statistical Analysis

Statistical analyses were performed using IBM SPSS Statistics 20.0 (IBM, Armonk, NY, USA). The normality of data distribution was assessed using the Kolmogorov–Smirnov and Shapiro–Wilk tests. Descriptive statistics were presented as mean ± standard deviation (SD), frequency, and percentage. For comparisons of CPQ8–10 subscale scores between groups, the Mann–Whitney U test was used for non-normally distributed data, whereas the independent samples t-test was used for normally distributed variables. The Chi-square test (χ^2^) was applied for categorical variables such as sex. For the comparison of children classified as regular versus irregular users of space maintainers, either the Mann–Whitney U test or the independent samples t-test was used, depending on data distribution.

To assess the main and interaction effects of group (space maintainer vs control) and sex on total CPQ8–10 scores, a two-way analysis of variance (ANOVA) was performed. Additionally, a one-way ANOVA was conducted to evaluate differences among age groups (8–9–10 years). A significance level of P < 0.05 was considered statistically significant.

## RESULTS

The mean age of children using space maintainers was 8.7 ± 0.76 years, whereas that of the control group was 8.9 ± 0.78 years. According to the Mann–Whitney U test, no significant difference in age was found between the groups (P = 0.189). However, a significant difference was observed in gender distribution (χ^2^ = 4.84, P = 0.028). Girls were more frequent in the control group (66.7%), whereas boys were predominant among space maintainer users (56.9%) (Table 1).

**Table 1:** Demographic characteristics of space maintainer users and healthy controls

Variable	Space maintainer users (n = 51)	Controls (n = 51)	P value
**Age (years, Mean ± SD)**	8.7 ± 0.76	8.9 ± 0.78	0.189
**Gender (n, %)**			
**– Girls**	22 (43.1%)	34 (66.7%)	0.028*
**– Boys**	29 (56.9%)	17 (33.3%)
Mann–Whitney U, χ^2^, *P < 0.05 = statistically significant difference

The oral symptom scores (median: 5, IQR: 3–6), functional limitation scores (median: 4, IQR: 2–7), emotional scores (median: 7, IQR: 4–10), and social scores (median: 10, IQR: 6–13) of children using space maintainers were higher than those of controls (oral symptoms: median: 3, IQR: 2–5; functional limitations: median: 2, IQR: 1–4; emotional: median: 3, IQR: 1–6; social: median: 2, IQR: 0–4) (all P < 0.05). Total CPQ8–10 scores were also greater in children with space maintainers (26.88 ± 11.94) compared with controls (12.24 ± 7.31) (t(82.83) = 7.47, P < 0.001) (Table 2).

**Table 2:** Comparison of CPQ8–10 subscale and total scores between space maintainer users and healthy controls

CPQ8-10 Subscale	Space maintainer users (n = 51)	controls (n = 51)	Test statistic	P value
**Oral symptoms**	Median 5 (IQR 3–6) Mean 4.78 ± 2.56	Median 3 (IQR 2–5) Mean 3.67 ± 2.46	Mann–Whitney U = 950.0	0.018*
**Functional limitations**	Median 4 (IQR 2–7) Mean 4.71 ± 3.35	Median 2 (IQR 1–4) Mean 2.33 ± 2.09	Mann–Whitney U = 730.0	< 0.001*
**Emotional well-being**	Median 7 (IQR 4–10) Mean 7.02 ± 4.49	Median 3 (IQR 1–6) Mean 3.59 ± 2.79	Mann–Whitney U = 697.0	< 0.001*
**Social well-being**	Median 10 (IQR 6–13) Mean 10.31 ± 5.77	Median 2 (IQR 0–4) Mean 2.75 ± 3.05	Mann–Whitney U = 263.5	< 0.001*
**Total CPQ8-10 score**	26.88 ± 11.94	12.24 ± 7.31	t = 7.47 (df = 82.83)†	< 0.001*
*P < 0.05 = statistically significant difference

When regular and irregular users of space maintainers were compared, no statistically significant differences were found in any CPQ8–10 subdomain (P > 0.05). Although the irregular-use group had slightly higher emotional and total CPQ8–10 scores, these differences were not significant (Table 3).

**Table 3:** Comparison of CPQ8–10 subscale and total scores between regular and irregular space maintainer users

Subscale	Regular use (n = 28)	Irregular use (n = 23)	Test	P value
Oral symptoms	4.71 ± 2.79	4.87 ± 2.32	Mann–Whitney U	0.730
Functional limitations	4.43 ± 2.94	5.04 ± 3.83	Mann–Whitney U	0.827
Social well-being	9.71 ± 5.46	11.04 ± 6.18	Mann–Whitney U	0.525
Emotional well-being	6.07 ± 3.73	8.17 ± 5.11	t-test	0.096
Total CPQ8–10 score	25.11 ± 10.57	29.04 ± 13.34	t-test	0.245
*P < 0.05 = statistically significant difference.

Two-way ANOVA was performed to examine the effects of group and gender on total CPQ8–10 scores. As previously demonstrated, the group effect was significant (P < 0.001). However, there was no significant main effect of gender (F = 3.51, P = 0.064) or group × gender interaction (F = 0.53, P = 0.467), indicating that the negative impact of space maintainer use on OHRQoL was similar for both boys and girls. Additionally, one-way ANOVA showed no significant difference in total CPQ8–10 scores among age groups (8–9–10 years) (F(3, 98) = 0.32, P = 0.815).

## DISCUSSION

The present study aimed to assess the impact of removable space maintainer (RSM) use on the oral-health-related quality of life (OHRQoL) of 8–10-year-old children using the CPQ8–10 questionnaire. The results demonstrated that children using RSMs had significantly higher total and subscale CPQ8–10 scores compared with caries-free peers without any appliances, indicating a negative impact of space maintainer use on OHRQoL. Specifically, significant differences were observed in all four subscales – oral symptoms, functional limitations, emotional well-being, and social well-being – suggesting that the effect of RSMs extends beyond physical discomfort to emotional and social domains as well. Consequently, the null hypothesis which stated that there would be no significant difference in OHRQoL between the groups was rejected.

Studies examining the relationship between intraoral appliances and quality of life generally report that appliances negatively affect OHRQoL at the beginning of treatment, but patients tend to adapt over time.^3,12,27^ Ojehanon et al^23^ indicated that individuals using either fixed or removable orthodontic appliances experienced reduced quality of life due to speaking and chewing difficulties initially; however, quality of life improved as treatment progressed. That study, however, included a wide age range (8–30 years), and the OHIP-14 scale used is not sufficiently sensitive for younger children. In contrast, the present study employed the age-specific CPQ8–10 instrument and evaluated only children using RSMs. Our findings demonstrated that space maintainer users had lower quality-of-life scores across all subdomains (oral symptoms, functional limitations, emotional and social well-being) compared with controls. This difference may be attributed to the difficulty younger children experience in adapting to removable appliances. Despite the presence of children with up to three restored teeth in the control group and the potential for similar differences in quality of life between groups, the findings suggest that the impact of appliance use on oral-health-related quality of life should not be underestimated.

Similarly, Abutaleb et al^2^ reported that removable functional appliances such as Twin-Block negatively influenced OHRQoL at the beginning of treatment, particularly in socio-emotional domains. These results suggest that removable appliances require an adaptation process and may cause notable psychosocial impact in children. Although Twin-Block appliances and RSMs differ in terms of clinical indication, both treatments involve removable devices; thus, similar adaptation challenges may occur. Furthermore, because Twin-Block appliances may offer visible aesthetic improvements, social acceptance might be easier; in contrast, space maintainers do not provide aesthetic benefits, which may contribute to the more pronounced emotional and social impact observed in younger children.

Costa et al,^13^ evaluating 579 Brazilian children aged 11–14 years using fixed orthodontic appliances, found significantly higher age-specific OHRQoL scores in appliance users. The most pronounced differences were seen in functional limitations, emotional well-being, and social well-being. Despite differences in appliance type and age range, both studies support that the use of dental appliances is associated with a lower quality of life compared with that of children without appliances. Similarly, in a study^8^ using a modified version of the Health-Related Quality of Life (HRQoL) questionnaire, no statistically significant differences in oral-health-related quality of life were reported according to gender or type of orthodontic appliance among patients using fixed or removable orthodontic appliances, suggesting that the negative impact on OHRQoL may be more closely related to the presence of an appliance itself rather than to specific demographic factors or appliance types. Despite the observed difference in gender distribution between the space maintainers and the control groups, the findings of this study are consistent with existing literature, which demonstrates^8^ that gender does not significantly influence oral-health-related quality of life.

Although RSMs are primarily applied as a preventive intervention, the psychosocial burden they may impose should not be overlooked. Our findings suggest that in younger children (ages 8–10), this treatment can create not only functional challenges but also emotional and social stress. Therefore, paediatric dentists should inform families about the potential adaptation process prior to treatment and evaluate not only the clinical functionality of the appliance during follow-up visits but also the child’s psychosocial adaptation. Additionally, motivational approaches and child-friendly educational strategies may enhance treatment acceptance and satisfaction.

When CPQ8–10 subscale scores were compared according to the regularity of space maintainer use, irregular users demonstrated numerically higher scores across all subdomains, including oral symptoms, functional limitations, emotional well-being, and social well-being, as well as in the total CPQ8–10 score. Although these differences did not reach statistical significance, the consistently higher scores observed among irregular users may indicate greater difficulties in adapting to RSMs. In particular, the higher emotional well-being scores suggest that dental anxiety, discomfort, or frustration related to appliance use, as well as family-related factors such as inconsistent supervision or motivation, may negatively affect children’s perceived quality of life.

Previous oral-health-related quality of life (OHRQoL) research has predominantly focused on fixed orthodontic appliances or removable functional appliances, whereas RSMs remain underrepresented in the literature. The present cross-sectional study addresses this gap by evaluating children aged 8–10 years exclusively using RSMs with an age-appropriate instrument (CPQ8–10), thereby providing age-specific and appliance-specific evidence regarding the impact of these appliances on OHRQoL.

The main limitation of this study is its cross-sectional design, which does not allow for causal inference. Moreover, the lack of longitudinal follow-up prevents the assessment of changes in adaptability and OHRQoL over time. Although the control group consisted of children without active dental caries, a small degree of past restorative experience was permitted, which may have had a minor influence on baseline OHRQoL and should be acknowledged as a limitation. Another limitation of this study is that the regularity of removable space maintainer use was assessed based on self-reports from children and their parents, which may introduce a degree of subjectivity. Future research should incorporate longitudinal designs to better evaluate the effect of treatment duration and adaptation on OHRQoL in children wearing space maintainers.

## CONCLUSION

This study demonstrated that children aged 8–10 years who use RSMs have significantly lower oral-health-related quality of life compared with caries-free peers. The differences observed across all CPQ8–10 subscales indicate that RSMs affect not only functional limitations but also emotional and social well-being. From a clinical perspective, these findings highlight the importance of structured follow-up protocols for children using RSMs, particularly during the early stages of treatment. Regular follow-up visits focusing not only on appliance fit and function but also on the child’s emotional and social adaptation may support treatment acceptance. In addition, family-centred counselling, clear behavioural guidance, and age-appropriate motivational strategies may help reduce anxiety, improve compliance, and facilitate successful adaptation to space maintainers.
